# Fibrosing mediastinitis with pulmonary hypertension as a complication of pulmonary vein stenosis: A case report and review of the literature

**DOI:** 10.1097/MD.0000000000009694

**Published:** 2018-01-26

**Authors:** Yidan Li, Xiangli Meng, Yidan Wang, Yuanhua Yang, Xiuzhang Lu

**Affiliations:** a Department of Echocardiography, Heart Center; b Department of Respiratory and Critical Care Medicine, Beijing Chao Yang Hospital, Capital Medical University, Beijing, China.

**Keywords:** compression of pulmonary veins, fibrosingmediastinitis, pulmonary hypertension

## Abstract

**Introduction:**

Fibrosingmediastinitis (FM) is caused by a proliferation of fibrous tissue in the mediastinum encasing the mediastinal viscera that results in compression of mediastinal bronchovascular structures. Pulmonary hypertension (PH) is a severe complication of FM caused by extrinsic compression of pulmonary blood vessels.

**Case Presentation:**

Here, we present the case of a 47-year-old man who presented with a 10-year history of progressive hemoptysis and a 2-year history of shortness of breath, in whom a diagnosis of FM was made. Occlusion of the superior pulmonary veins was noted, with stenosis of the inferior pulmonary veins, leading to PH. Because the patient was a poor candidate for interventional catheterization, the preferred treatment for FM, his PH has been managed with diuretics, and he remains stable.

**Conclusions:**

FM is a serious, potentially life-threatening condition that is best managed in specialized centers.

## 1 Introduction

Fibrosingmediastinitis (FM), also known as collagenosis or sclerosing mediastinitis,^[1]^ is a rare but fatal disease with few therapeutic options. The causes of FM include mediastinal exposure to the *Histoplasma capsulatum* antigen, which induces an intense inflammatory response, resulting in proliferation and invasion of fibrous tissue into vital structures.^[2,3]^ Narrowing or even occlusion of the major pulmonary veins (PVs) is an uncommon but insidious vascular complication of FM.^[4]^ Diagnosis of PV stenosis is challenging due to the gradual onset of nonspecific symptoms, including fatigue and dyspnea, leading to delayed recognition of illness. In the late stages of FM, recurrent episodes of pulmonary edema and hemoptysis can occur, eventually becoming fatal.^[5–7]^

In this study, we report a case of pulmonary hypertension (PH) caused by FM that caused compression of the PVs. The patient provided written informed consent for the publication of this report.

## 2 Case report

A 47-year-old man presented with a history of a gradual worsening hemoptysis over more than a decade, with shortness of breath for the most recent 2 years. Two years prior, he had been diagnosed with pulmonary tuberculosis and was treated with medications for tuberculosis for 6 years without significant improvement. His past medical history, family history, and social history were noncontributory. His physical examination findings were normal. His complete blood count, comprehensive metabolic panel, C-reactive protein, erythrocyte sedimentation rate, HIV testing, and urinalysis yielded mostly normal results, except for an elevated erythrocyte sedimentation rate (22 mm/h) and D-dimer concentration (1137.86 mg/L).

**Figure 1:**
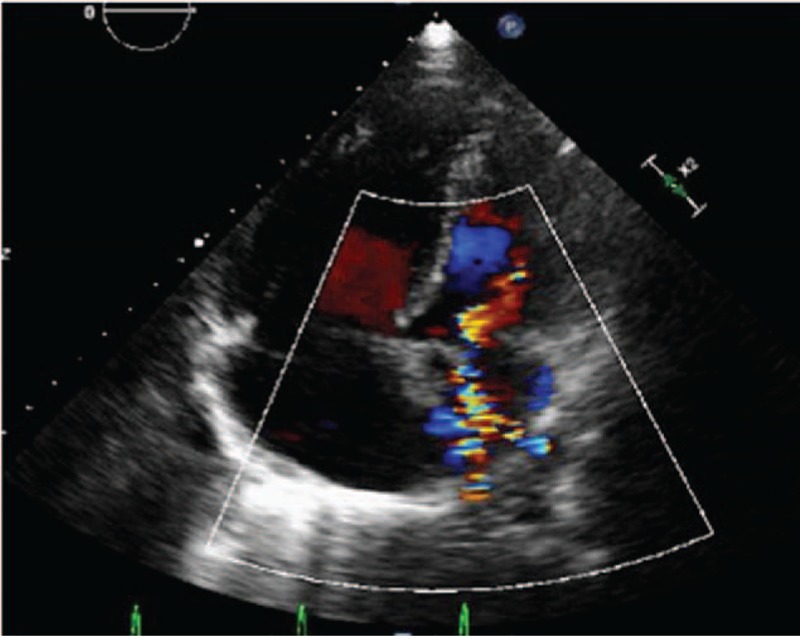
Four-chamber echocardiography view of PV stenosis in patient with FM. Note the presence of turbulent flow within the left atrium.

**Figure 2:**
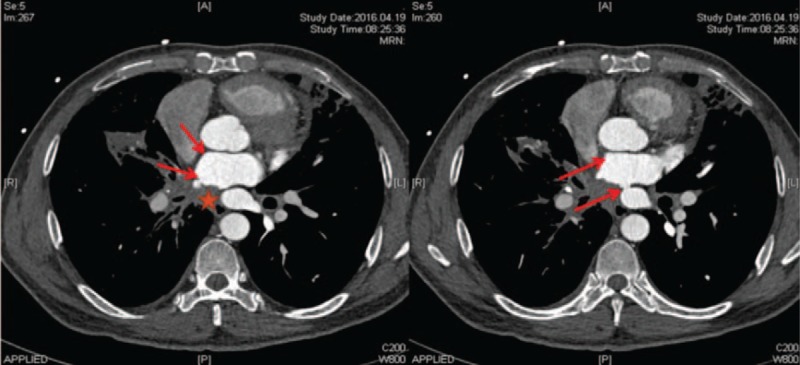
Chest CT (mediastinal window) in patient with FM. Multiple soft-tissue shadows (asterisk) are evident. Left and right superior PV occlusion and inferior PV stenosis were observed (arrows).

**Figure 3:**
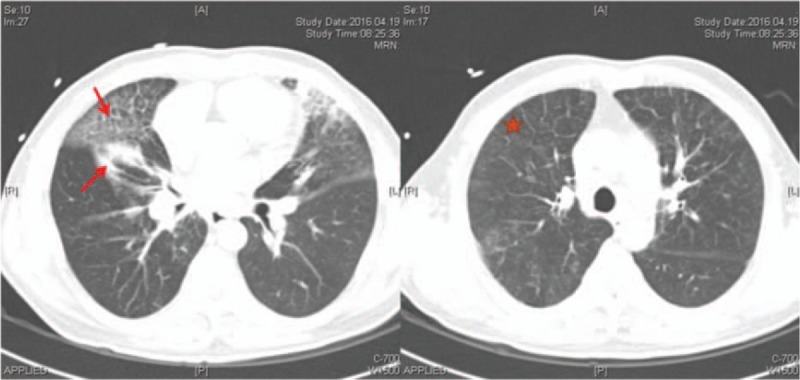
Chest CT (pulmonary window) in patient with MT. Note the findings of pulmonary consolidation (arrow) and pulmonary edema (asterisk).

Echocardiography results were consistent with PH and right ventricular dysfunction, with an estimated pulmonary arterial systolic pressure of 78 mm Hg. The echocardiogram also identified turbulent blood flow within the left atrium (Video 1). Color Doppler demonstrated 2 high-velocity continuous jets originating from the right and left PVs, indicative of PV stenosis (Fig. 1). A ventilation/perfusion scan demonstrated diffusely decreased perfusion to the right lung, most prominent in the right upper lobe. Contrast-enhanced computed tomography (CT) of the thorax revealed multiple soft-tissue shadows, mediastinal lymphadenopathy, and calcification, findings consistent with FM (Fig. 2). The CT also revealed bilateral patchy ground glass opacities suggestive of pulmonary edema with occlusion of the left and right superior PVs and stenosis of the inferior PVs (Figs. 2 and 3). In addition, pulmonary consolidation was observable (Fig. 3).

A right heart catheterization was performed, during which an increased PA pressure of 69/29 mm Hg (mean, 42 mm Hg) was observed with a wedge pressure of 16 mm Hg. His cardiac index was 3.52 L/min/m^2^. Cardiac angiography revealed no contrast flow into the left atrium through the left and right superior PVs; a small amount of contrast reached the left atrium through the left and right inferior PVs (Video 2). It also demonstrated superior PV occlusion and inferior PV stenosis. Together with the history and physical examination, these observations led to a clinical diagnosis of chronic FM with PV occlusion and stenosis leading to PH.

In a discussion of the potential treatment options, the surgeon suggested that efforts to strip away the fibrotic soft tissue could result in rupture of the PVs, and thus recommended a catheter-based intervention for the PV stenosis. However, the interventional radiologist thought that endovascular stenting of the stenotic lesions was likely to result in recurrent stenosis. After comprehensive consideration of the risks of surgery and a catheter-based intervention, the patient chose to proceed with conservative management and was discharged with a diuretic therapy plan aimed at reducing his pulmonary edema.

In September 2016, he was admitted to the hospital for a pulmonary infection and was discharged after showing improvement. At a follow-up visit in November 2016, the patient confirmed that he had adhered to the diuretic regimen, but felt no significant improvement in his symptoms. At present, his condition is stable.

## 3 Discussion

PH is a progressive, severe hemodynamic disorder that has the potential to be fatal if left untreated. The World Health Organization classification of PH lists FM as group 5: “other” because of its unclear or multifactorial pathophysiology.^[8]^ FM is a rare, often benign, but potentially lethal disorder caused by proliferating fibrous tissue in the mediastinum that encases the mediastinal viscera, causing extrinsic compression of mediastinal bronchovascular structures, ultimately presenting as a progressive, insidious disease with a variable natural history.^[9]^ Usually, FM is associated with a history of granulomatous disease, such as sarcoidosis, tuberculosis, or histoplasmosis.^[10–12]^ Possible association with fungal (e.g., aspergillosis, blastomycosis, mucormycosis, or cryptococcosis) or round worm parasite (*Wuchereriabancrofti*) infections have also been reported.^[13–17]^ Autoimmune diseases (including rheumatoid arthritis and systemic lupus erythematosus),^[14]^ Behçetdisease,^[16]^ mediastinalradiation therapy,^[14,18]^ and methysergide therapy^[19]^ can also induce FM. Moreover, anidiopathic form of FM without any known triggering factor has been described.^[20,21]^

Cough, chest pain, and dyspnea are the main symptoms of FM, though the clinical manifestations of FM depend largely on which structures of the mediastinum are affected.^[22]^ Typical complications include airway compression, which can lead to postobstructive pneumonia or atelectasis, bronchial erosion by calcific lymph nodes, esophageal compression, or compression of pulmonary arteries and/or veins.^[23,24]^ Patients with pulmonary vessel compression can develop hemoptysis due to bronchial artery hypertrophy, which is frequently observed in FM.^[11,14]^

Diagnosis of FM can be challenging in the context of PH because pulmonary artery compression, PH, and right heart failure are considered to be important causes of morbidity and mortality in FM.^[4,25]^ Most cases of PV occlusion have been reported from autopsy studies, indicating the difficulty of making the diagnosis of FM antemortem.^[26]^ Imaging studies and a suggestive clinical context are usually sufficient to confirm the diagnosis. The presence and severity of PV stenosis can be assessed noninvasively by echocardiography, CT angiography, radionuclide perfusion imaging, or magnetic resonance imaging.^[27–31]^ PV stenosis is associated with reduced PV diameters with increased flow velocities and turbulent flow signals on color-flow Doppler.^[27–29]^ Although definitive diagnostic criteria are not available, peak flow velocities exceeding 80 or 110 cm/s^[27]^ have been proposed as criterion to differentiate stenotic from normal PV flow. However, higher peak velocities can occur in PVs without marked stenosis.^[31]^ Combining increased peak velocities and a turbulent flow pattern may improve diagnostic accuracy.^[27,30]^

Success with stenting of congenital PV stenosis has led to percutaneous stenting of FM-related pulmonary vessels, which is currently the only standard treatment option for FM. However, stenting is challenging and should be performed by centers equipped to provide comprehensive evaluation of the condition and to perform complex and risky percutaneous procedures that may include transatrial septalpuncture.^[32]^ Appropriate patient selection and identification of the vascular territory in which to intervene are paramount. It is critical that corresponding ventilation be preserved, and the end-result should be an intact arterial-venous relationship within the lung region. PH can improve considerably, especially when proximal pulmonary arteries are involved, and in such cases, symptoms lessen dramatically.^[32]^ However, the durability of PV stents is suboptimal because stents are susceptible to early and late restenosis and often require reintervention within 1 to2 years.^[33,34]^ Recurrent stenosis is the most common complication to arise after PV stenting. Additional risks associated with PV intervention include hemoptysis, severe pulmonary hemorrhage, dissection,^[10]^ PV rupture,^[8,35]^ and embolic events.^[30]^ Prieto et al^[36]^ published the largest series, to our knowledge, of PV interventions after PV isolation (34 patients; 55 PVs). Postoperative restenosis rates after 25 months were 72% for angioplasty and 33% for stenting.^[36]^ Compared to dilation, patency rates were greater with stents, particularly with stents ≥10 mm in diameter.^[36]^ PV stenosis due to FM remains a condition with significant morbidity but few treatment options. Further innovative therapies are needed to provide better treatment options for this high-risk patient population.

Our patient had both superior PV obstruction and inferior PV stenosis, impeding blood flow to the left atrium and underlying his clinical findings, especially pulmonary edema. The elevated pulmonary artery wedge pressure suggested a post capillary location of the obstruction. PH was possibly the result of a longstanding high wedge pressure. Both superior PVs were completely occluded, with significant stenosis in both inferior PVs. However, gradual obstruction of the PVs from any cause can lead to an unusual form of interstitial fibrosis in the lung parenchyma drained by the obstructed veins. This peculiar form of pulmonary fibrosis results from extreme pulmonary venous hypertension. PH can ensue if enough central veins are affected by the underlying fibroproliferative process; histopathology in such cases reveals thickening of the intimal, medial, and adventitial layers of the PVs.

In conclusion, FM involving the pulmonary vessels should be included in the differential diagnosis of patients with cough, progressive respiratory distress, and recurrent hemoptysis, or even in that of patients with unexplained pulmonary interstitial fibrosis. Additional findings in patients with FM may include pulmonary edema or local pulmonary congestion; transesophageal echocardiography could aid in making the diagnosis. Endovascular stenting can bring symptom relief, but this treatment modality may be complicated by restenosis. As illustrated by this case, PV stenosis, with or without interventions, requires long-term follow-up because the disease may evolve or recur.
